# Interleukin-6 stimulates Akt and p38 MAPK phosphorylation and fibroblast migration in non-diabetic but not diabetic mice

**DOI:** 10.1371/journal.pone.0178232

**Published:** 2017-05-23

**Authors:** Tsubame Nishikai-Yan Shen, Shigeyuki Kanazawa, Makiko Kado, Kayoko Okada, Lin Luo, Ayato Hayashi, Hiroshi Mizuno, Rica Tanaka

**Affiliations:** 1 Department of Plastic and Reconstructive Surgery, Juntendo University School of Medicine, Tokyo, Japan; 2 Department of Plastic Surgery, Osaka University Graduate School of Medicine, Osaka, Japan; University of New Mexico Health Sciences Center, UNITED STATES

## Abstract

Persistent inflammatory environment and abnormal macrophage activation are characteristics of chronic diabetic wounds. Here, we attempted to characterize the differences in macrophage activation and temporal variations in cytokine expression in diabetic and non-diabetic wounds, with a focus on interleukin (IL)-6 mRNA expression and the p38 MAPK and PI3K/Akt signaling pathways. Cutaneous wound closure, CD68- and arginase-1 (Arg-1)-expressing macrophages, and cytokine mRNA expression were examined in non-diabetic and streptozotocin-induced type 1 diabetic mice at different time points after injury. The effect of IL-6 on p38 MAPK and Akt phosphorylation was investigated, and an *in vitro* scratch assay was performed to determine the role of IL-6 in primary skin fibroblast migration. Before injury, mRNA expression levels of the inflammatory markers iNOS, IL-6, and TNF-α were higher in diabetic mice; however, IL-6 expression was significantly lower 6 h post injury in diabetic wounds than that in non-diabetic wounds. Non-diabetic wounds exhibited increased p38 MAPK and Akt phosphorylation; however, no such increase was found in diabetic wounds. In fibroblasts from non-diabetic mice, IL-6 increased the phosphorylation of p38 MAPK and levels of its downstream factor CREB, and also significantly increased Akt phosphorylation and levels of its upstream factor P13K. These effects of IL-6 were not detected in fibroblasts derived from the diabetic mice. In scratch assays, IL-6 stimulated the migration of primary cultured skin fibroblasts from the non-diabetic mice, and the inhibition of p38 MAPK was found to markedly suppress IL-6–stimulated fibroblast migration. These findings underscore the critical differences between diabetic and non-diabetic wounds in terms of macrophage activation, cytokine mRNA expression profile, and involvement of the IL-6-stimulated p38 MAPK–Akt signaling pathway. Aberrant macrophage activation and abnormalities in the cytokine mRNA expression profile during different phases of wound healing should be addressed when designing effective therapeutic modalities for refractory diabetic wounds.

## Introduction

Approximately 2.8% of the world population is reported to be affected by diabetes, and approximately 15% of patients with diabetes have impaired cutaneous wound healing, which poses a serious risk of limb amputation and compromised quality of life [1, 2]. Wound healing is a complex series of spatially and temporally coordinated dynamic events, involving hemostasis, inflammation, proliferation, and remodeling phases. A critical issue in non-healing diabetic wounds is a prolonged phase of inflammation and neutrophil infiltration, characterized by an abundance of pro-inflammatory macrophages, cytokines, and proteases. Macrophages are key modulators of host defense, wound healing, and immune regulation [3]. They are involved in distinct immune functions such as inflammation and tissue repair, and are classified into two distinct phenotypes: classically activated macrophages (M1) and alternatively activated macrophages (M2). The M1 phenotype secretes pro-inflammatory cytokines and chemokines, toxic intermediates, and reactive oxygen intermediates, whereas the M2 phenotype is anti-inflammatory and involved in tissue repair and remodeling [4–11]. In non-diabetic wounds, the M1 phenotype appears in the initial stage of wound healing, followed by the M2 phenotype at later stages. Diabetic wounds, however, exhibit abnormal macrophage activation, showing insufficient M1 in the early stage and delayed activation of M2 [12, 13]. Macrophages are a major source of cytokines in wounds, and their dysfunction is known to be a factor in the pathogenesis of chronic wounds in diabetes [14–17]. Identifying the factors associated with macrophage dysfunction and cytokine dysregulation is therefore crucial for preventing wounds from becoming arrested at the inflammatory stage, as well as for promoting the healing of diabetic wounds [11, 18, 19].

Cytokines are known to transduce downstream signals via different signaling pathways [20]. Two such important pathways are those of the protein kinase PI3K/Akt and the stress-activated protein kinase p38 MAPK. The PI3K/Akt signaling pathway is involved in diverse cellular functions and has been associated with fibroblast migration and proliferation in wound healing [21, 22]. Although the role of the p38 MAPK signaling pathway in wound healing is not yet established, recent studies suggest its involvement in cellular migration in wounds [23].

IL-6 is a crucial inflammatory cytokine in the early stages of wound healing; however, it is also reported to be present in high abundance in chronic wounds [24]. The abundance of IL-6 implies involvement of the M1 phenotype and persistent inflammation in chronic wounds [17, 19, 25, 26]. There is overwhelming evidence highlighting the critical role of IL-6 in facilitating wound healing [19, 27, 28]; however, not much information is available on the temporal variations in IL-6 expression in diabetic wounds or on the involvement of p38 MAPK or PI3K/Akt signaling pathways in diabetes. We hypothesized that abnormal IL-6 mRNA expression, macrophage activation, and p38 MAPK and PI3K/Akt signaling contribute to impaired wound healing in diabetes.

This study aimed to investigate macrophage activation, temporal variations in the mRNA expression of cytokines, and the phosphorylation of Akt and p38 MAPK in diabetic and non-diabetic wounds. Efforts were also made to elucidate the effect of IL-6 on fibroblast migration and on the stimulation of p38 MAPK or PI3K/Akt signaling pathways in diabetic and non-diabetic wounds.

## Materials and methods

This study was approved by the Ethical Animal Care and Use committee of Juntendo University School of Medicine (permit number, 260136).

### Diabetic mouse model

C57BL/6J male mice (age: 10 weeks, weight: 20–25 g) were purchased from CLEA Japan (Kawasaki, Japan). Intraperitoneal injections of 50 mg/kg streptozotocin (STZ) in 50 mmol/L sodium citrate buffer (pH 4.5) were administered to mice for 5 consecutive days to obliterate pancreatic β cells. Mice maintaining fasting glucose levels of 200 mg/dL for at least 4 weeks before wounding were considered diabetic. Non-diabetic mice received intraperitoneal injections at the same time points with an equal volume of 50 mmol/L sodium citrate buffer. In total, 48 mice were used in this experiment (n = 4 per group). Mice were inhalation-anesthetized with 2% isoflurane to completely alleviate suffering. At the end of the experiment, mice were euthanized with a high concentration of isoflurane.

### Wound model

We used euglycemic and diabetic C57BL/6J male mice. Each mouse was anesthetized and depilated, and a set of bilateral 6-mm punch biopsy excisions was made on the dorsum to yield full-thickness wounds, including the hypodermis and panniculus. India ink was applied intradermally at the margins to permanently mark the wound edge. A silicone stent (Grace Bio-Laboratories, Bend, OR, USA) with an inner diameter of 8 mm was sutured with 5–0 nylon (Ethicon, Somerville, NJ, USA) around each wound to minimize skin contracture and to ensure healing by secondary intention. Wounds were photographed with a DSC-T900 camera (Sony, Tokyo, Japan) from a distance of 6.5 cm, with the lens oriented parallel to the wound. The wound area was measured using Photoshop CS3 (Adobe Systems, San Jose, CA, USA). The internal diameter of the silicone stent was used for calibration to correct for magnification, perspective, or parallax effects. The percent wound closure [(1 − (wound area/original wound area)) × 100] was measured photogrammetrically on days 0, 0.25, 1, 3, 5, 7, and 9. Wounds were harvested from sacrificed animals on postoperative days 0, 0.25, 1, 3, 5, 7, and 9 (n = 4 per group at each time point). A full-thickness excision (3 mm beyond the margin of the wound edge) was made. One-half of the wound was kept in liquid nitrogen for subsequent PCR and western blotting.

### Reagents

Primary antibodies against Akt, p38 mitogen-activated protein kinase (MAPK), cAMP response element–binding protein (CREB), phosphoinositide 3-kinase (PI3K), p-Akt (Ser473), p-p38 MAPK (Thr180/Tyr182), p-CREB (Ser133), and p-PI3K (Tyr458/p55 (Tyr199) were purchased from Cell Signaling Technology (MA, USA). Anti-GAPDH was from Santa Cruz Biotechnology Inc. (TX, USA). Anti-rabbit HRP-linked secondary antibody and anti-mouse HRP-linked secondary antibody were purchased from Cell Signaling Technology. STZ and mouse IL-6 were obtained from Sigma-Aldrich (MO, USA), and SB203580 was sourced from Calbiochem (CA, USA).

### Immunofluorescence

Dorsal wound skin samples were fixed in 4% paraformaldehyde overnight. This was followed by treatment of the skin samples with 5–30% sucrose/phosphate-buffered saline (PBS) solution overnight, embedding in tissue processing medium (OCT), and storage at −80°C. Frozen wound tissue sections were incubated with PBS containing 10% goat serum. Then, the sections were further incubated overnight with a combination of anti-CD68 and anti-arginase 1 (Arg-1) antibodies (Abcam Inc., Cambridge, MA, USA) at 4°C. All antibodies were used at a dilution of 1:100. Tissue sections were incubated with Alexa 586–conjugated goat anti-rat antibody and Alexa 488–conjugated goat anti-rabbit antibody (1:200; Molecular Probes, Eugene, OR, USA) at room temperature for 60 min and mounted with Hoechst 33342 for nuclear staining. Negative controls without primary antibodies were used in each case to rule out nonspecific labeling (S1 and S2 Figs). For positive staining, we used liver tissue for arginase staining and spleen tissue for CD68 staining. The wound edge was analyzed using a LSM 510 two-photon laser confocal scanning system (Zeiss, Thornwood, NY, USA).

### Cell culture

Primary skin fibroblasts (non-diabetic fibroblasts) were derived from mice aged 19–20 weeks and cultured in Dulbecco’s modified Eagle’s medium (DMEM) containing 10% fetal bovine serum (FBS), and fibroblasts from mice with type 1 diabetes (diabetic fibroblasts) were cultured in high-glucose DMEM (DMEM with 4.5 g/L glucose), 100 U/mL penicillin, and 100 mg/mL streptomycin in a humidified incubator at 37°C with a 5% CO_2_ atmosphere. Cells between passages 2 and 3 were used in the experiments.

### Mouse wound healing assay

Confluent cells were cultured in 60-mm dishes (Becton Dickinson, NJ, USA). The cells were serum-starved in DMEM for 16 h and then wounded with linear scratches (500 mm) by a sterile pipette tip. The initial area devoid of cells was marked and quantified on images obtained at the baseline, and treatments with IL-6 (10 ng/mL) or SB203580 (20 μM) were initiated in DMEM [27]. Cells were imaged under an inverted phase-contrast microscope (Nikon Eclipse Ti-u; Nikon Corporation, Japan) equipped with a Nikon SD-Fi2 camera. The area devoid of cells was quantified using Adobe ImageReady CS2 software. The proportion of the initial area devoid of cells to that occupied by cells after 0–24 h of treatment was expressed as the degree of migration.

### Western blot analysis

Whole-cell lysates and whole-skin lysates were subjected to SDS-PAGE, and the proteins that migrated were electrically transferred to a polyvinylidene fluoride (PVDF) microporous membrane (Life Technologies, MA, USA). The membrane was incubated at 4°C for 18 h with antibodies against the following proteins: p-Akt, Akt, p-p38 MAPK, p38 MAPK, p-CREB, CREB, p-PI3K, PI3K (1:1000), and GAPDH (1:2000). After incubation, anti-mouse HRP-linked secondary antibody (1:2000, for GAPDH) or anti-rabbit IgG HRP (1:2000, for p-Akt, Akt, p-p38 MAPK, p38 MAPK, p-CREB, CREB, p-PI3K, and PI3K) was added, and the membrane was incubated for 30 min at room temperature. The chemiluminescence of antigenic proteins on the membrane was monitored using the Lumi-LightPLUS western blotting kit (Roche Diagnostics Co., Basel, Switzerland), and the images were analyzed using an image analyzer (LAS-3000; Fujifilm Co., Tokyo, Japan).

### RNA isolation and RT-PCR

Total RNA was extracted from skin, using TRIzol (Ambion RNA, Life Technologies). Real-time RT-PCR was performed using the fluorescent dye SYBR Green I and the SYBR Premix Ex Taq kit (Perfect Real Time; Takara Bio, Shiga, Japan) in a StepOne PCR system (Life Technologies, CA, USA). Information available from GenBank was used to design the primers (IL-1, XM 006498795, IL-4, NM_021283, IL-6, NM_031168, IL-10, NM_010548, iNOS, NM_010927, TNFα, AB023024, TGFβ, NM_009370, arginase, U51805). The primers were synthesized by Life Technologies (Table 1).

**Table 1:** Primer sequences used for real-time PCR.

Marker	Forward and reverse sequences
IL-1	5′- ATTAGGCAGCACTCTCTAGAACAGA -3′ and 5′-TTCCTGTGCAAACTCTAAGAGAAGT-3′;
IL-4	5′-TAGTTGTCATCCTGCTCTTCTTTCT-3′ and 5′-GATCTCTCTCAAGTGATTTTTGTCG-3′
IL-6	5′-GTTGCCTTCTTGGGACTGATG-3′ and 5′-TGGGAGTGGTATCCTCTGTGAA-3′;
IL-10	5′-ATCTTAGCTAACGGAAACAACTCCT-3′ and 5′- TAGAATGGGAACTGAGGTATCAGAG-3′
iNOS	5′-GGCAGCCTGTGAGACCTTTG-3′ and 5′- CGTTTCGGGATCTGAATGTGA-3′
TNF-α	5′-ACCCTCACACTCAGATCATCTTC-3′ and 5′-TGGTGGTTTGCTACGACGT-3′
TGF-β	5′-GAGATTCCAGCTGTTGTTCTGTTAT-3′ and 5′-CTGTACTGCACTCCCAAACTATTCT-3′
Arginase	5′-CTCCAAGCCAAAGTCCTTAGAG-3′ and 5′-AGGAGCTGTCATTAGGGACATC-3

### Statistical analysis

All data are expressed as the mean ± SE. The statistical difference between two groups was analyzed by Student’s *t*-test, and that among more than three groups by one-way analysis of variance (ANOVA) with Bonferroni multiple comparison tests. SPSS Software (IBM Software, Tokyo, Japan) was used for the analysis. p < 0.05 was considered statistically significant.

## Results

### Wound healing is delayed in diabetic mice

**Fig 1:**
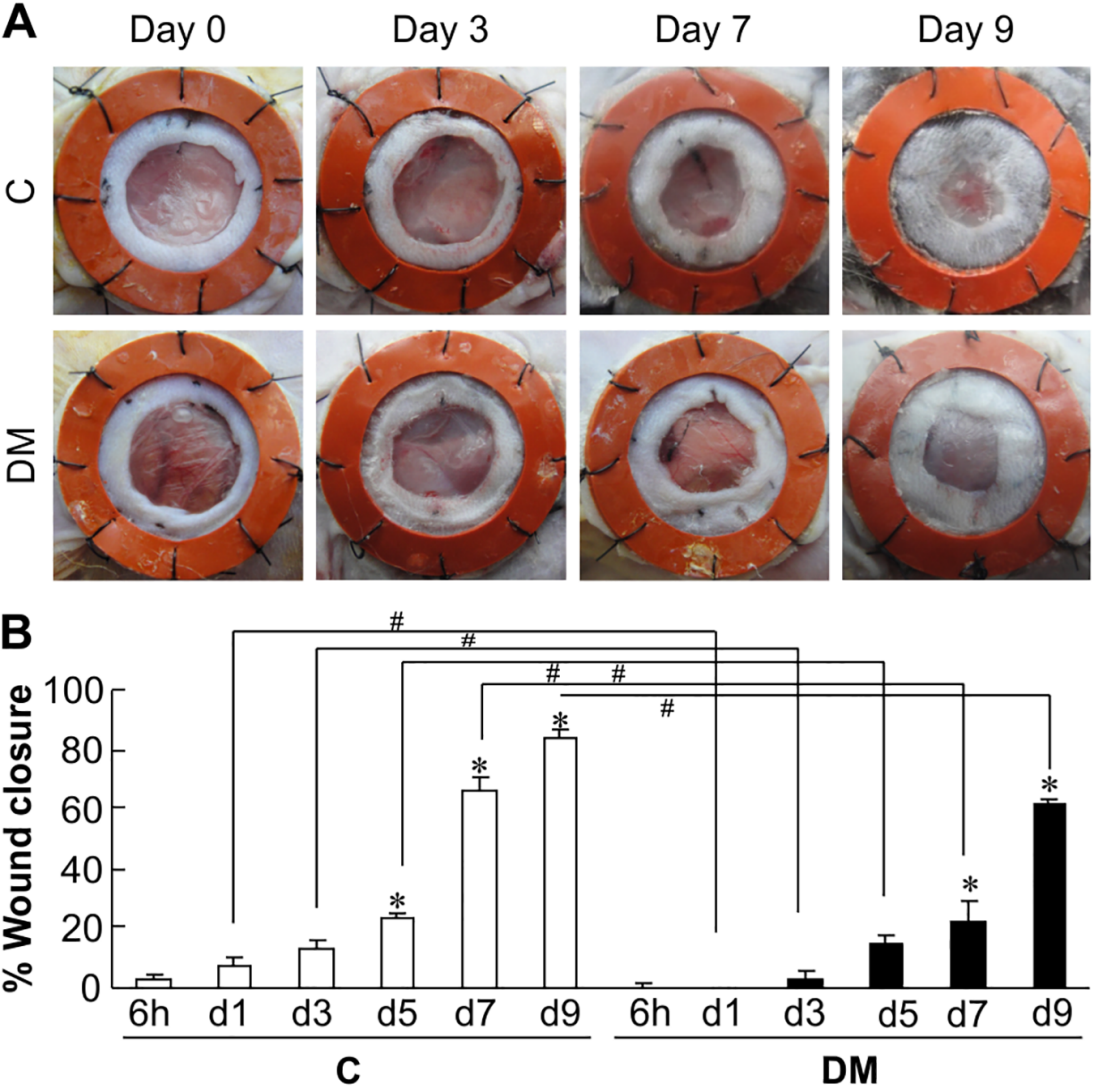
Representative images and graph showing wound healing in non-diabetic and diabetic mice. (A) Representative images of wounds photographed at the time points indicated between day 0 to day 9 in euglycemic mice (Con) and diabetic mice (DM). (B) The graphs show the comparison of percent wound closure between euglycemic mice and diabetic mice. The data represent the mean ± SE [n = 4, *p < 0.05 vs. Con (6 h), # p < 0.05 Con vs. DM].

Gross images of wound healing and % wound closure in diabetic and non-diabetic mice are shown in Fig 1A and 1B. On days 7 and 9 after injury, % wound closure significantly improved in non-diabetic mice (65.9% ± 4.5% and 83.8% ± 2.6%, respectively) (Fig 1B). Diabetic mice, however, showed significantly lower wound closure than non-diabetic mice; on 7 and 9 days after injury, the percent wound closure values were just 22.5% and 61.1%, respectively, confirming delayed wound healing in diabetic mice. Furthermore, diabetic mice had significantly lower percent wound closure than the euglycemic mice at all the time points studied.

### Diabetic wounds display increased numbers of CD68+ cells and delayed anti-inflammation phase

**Fig 2:**
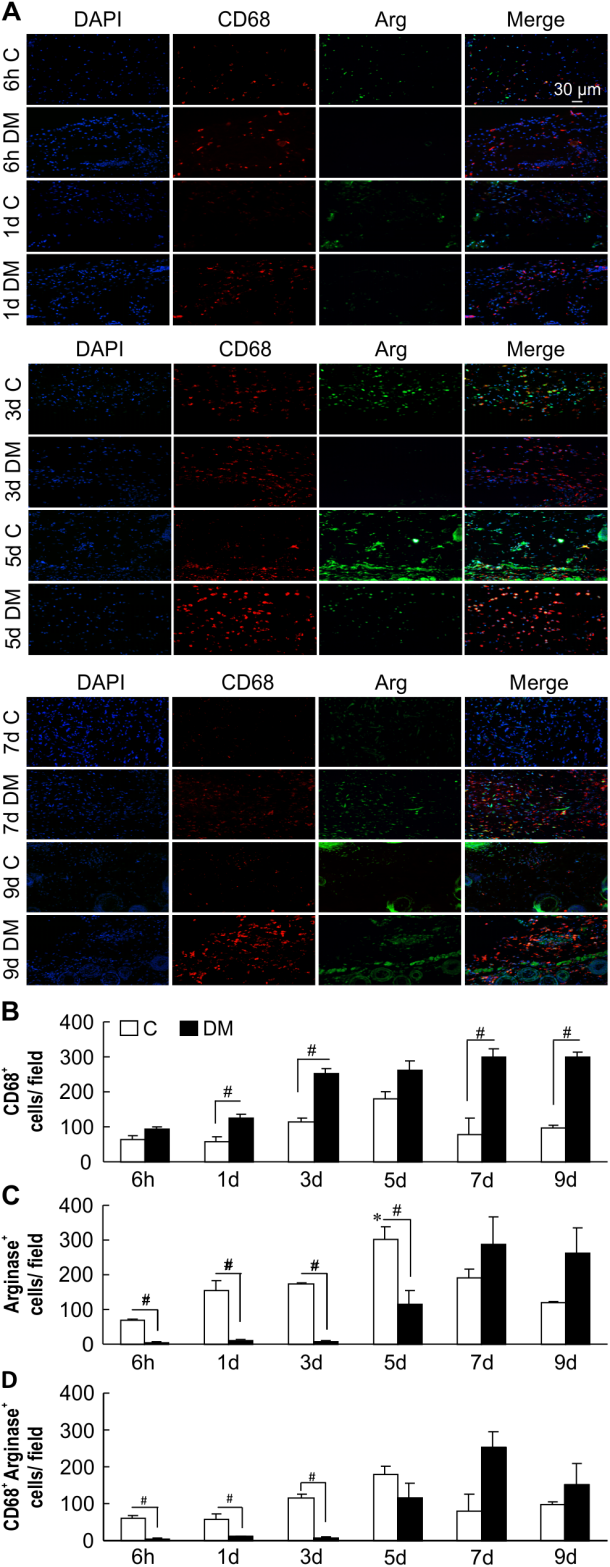
Identification of macrophages in non-diabetic and diabetic wounds. (A) Representative immunostaining for CD68 (red) and Arg-1 (green). Nuclear counterstaining was performed using DAPI (blue). Scale bar = 30 μm. Non-diabetic wound macrophages on days 3 and 5 after wounding stained positive for Arg-1. CD68 expression at almost all time points increased in diabetic wounds compared with that in non-diabetic wounds. (B) and (C) quantitation of CD68- and Arg-1single positive cells (D) quantitation of CD68- and Arg-1double positive cells The data represent the mean ± SE [n = 3, *p < 0.05 vs. Con (6 h), # comparison between two groups p < 0.05)].

To identify wound-associated macrophages and macrophage polarization status, we performed immunofluorescence staining for the macrophage marker CD68 and M2 marker Arg-1 in samples from non-diabetic and diabetic wounds [29, 30]. There were more CD68-positive macrophages (red) in diabetic wounds than in non-diabetic wounds at the corresponding time points (Fig 2A), indicating that although macrophages were present in both groups, there was a higher abundance of macrophages in diabetic wound skin. In non-diabetic wounds, the expression of Arg-1 (green) was highest 3–5 days after wounding, whereas in diabetic wounds, strong expression of Arg-1 was observed at approximately 7–9 days after wounding (Fig 2A). As shown in Fig 2B and 2C, although the number of CD68^+^ cells was significantly increased in DM mice, that of Arg-1^+^ cells was decreased. Furthermore, the number of CD68+Arg double-positive cells was significantly lower in diabetic mice until day 3 and begin to increase only after day 5 (Fig 2D). These data indicate that the appearance of the anti-inflammatory macrophage phenotype M2 is delayed in diabetic wounds.

### Expression of IL-6 mRNA is decreased in diabetic mouse skin immediately after wounding

**Fig 3:**
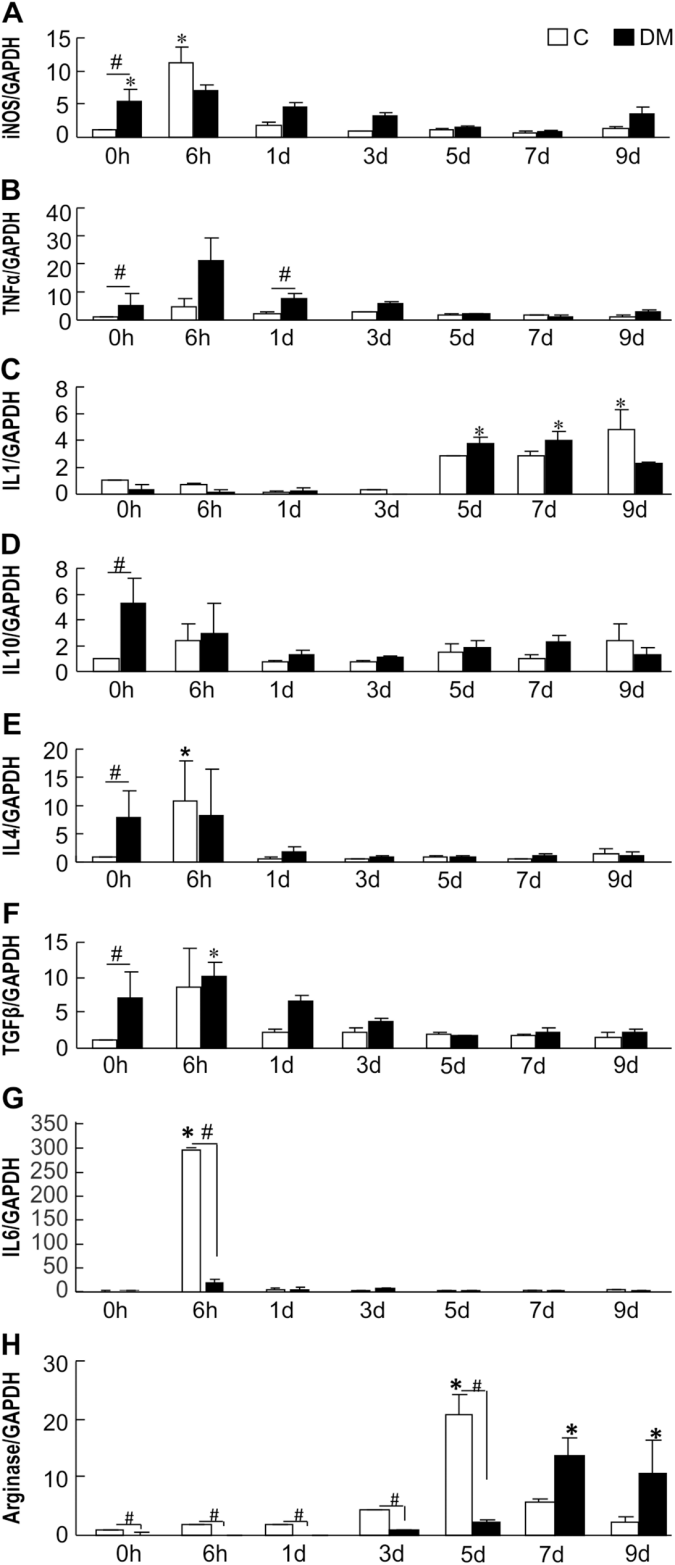
mRNA expression of M1 and M2 cytokines in non-diabetic and diabetic wounds. mRNA expression of cytokines in skin from non-diabetic mice and diabetic mice. The skin of the wound sites was retrieved at 0 h, 6 h, 1 day, 3 days, 5 days, 7 days, and 9 days. Proinflammatory mRNA expression was measured by real-time RT-PCR; (A) iNOS, (B) TNFα, (C) IL-1, (G) IL-6. Anti-inflammatory cytokine expression was measured by real-time RT-PCR; (D) IL-10, (E) IL-4, (F) TGFβ, (H) arginase. The data represent relative expression of each cytokine after normalization with GAPDH levels in mean ± SE (n = 3, *p < 0.05 vs. Con (0 h), # *p* < 0.05 Con vs DM).

To determine the effect of the cutaneous inflammatory environment on wound healing, we measured the expression of inflammatory cytokine mRNA in pre- and post-injury skin by real-time RT-PCR. Before wounding (at 0 h), the mRNA expression of iNOS, TNFα, and IL-6 was significantly higher in the skin of diabetic mice than in the skin of non-diabetic mice (Fig 3A, 3B and 3G; 0 h). These data correspond with persisting hyperglycemia–induced inflammatory alterations and perpetuation of chronic autoimmune responses in various tissues, including the skin [21, 31, 32]. Notably, however, the mRNA expression levels of IL-10, IL-4, and TGFβ were significantly elevated in diabetic mice compared to those in the non-diabetic mice before wounding (Fig 3D, 3E and 3F; 0 h). The non-diabetic mice showed significantly higher expression levels of Arg-1 mRNA at several time points as compared with diabetic mice: before injury (1.00 ± 0.00 vs. 0.19 ± 0.19), 6 h after injury (1.65 ± 0.16 vs. 0.01 ± 0.00), 1 day after injury (1.86 ± 0.10 vs. 0.13 ± 0.06), 3 days after injury (4.36 ± 0.15 vs. 0.92 ± 0.08), and 5 days after injury (20.75 ± 3.45 vs. 2.33 ± 0.40) (Fig 3H). Remarkably, non-diabetic wounds displayed significantly increased IL-6 expression 6 h after injury (Fig 3G). These findings therefore indicate immediate initiation of an inflammatory phase of wound healing in non-diabetic mice and the absence of a similar phenomenon in diabetic mice post injury [33].

### Wounding causes phosphorylation of Akt and p38 MAPK in non-diabetic but not diabetic wounds

**Fig 4:**
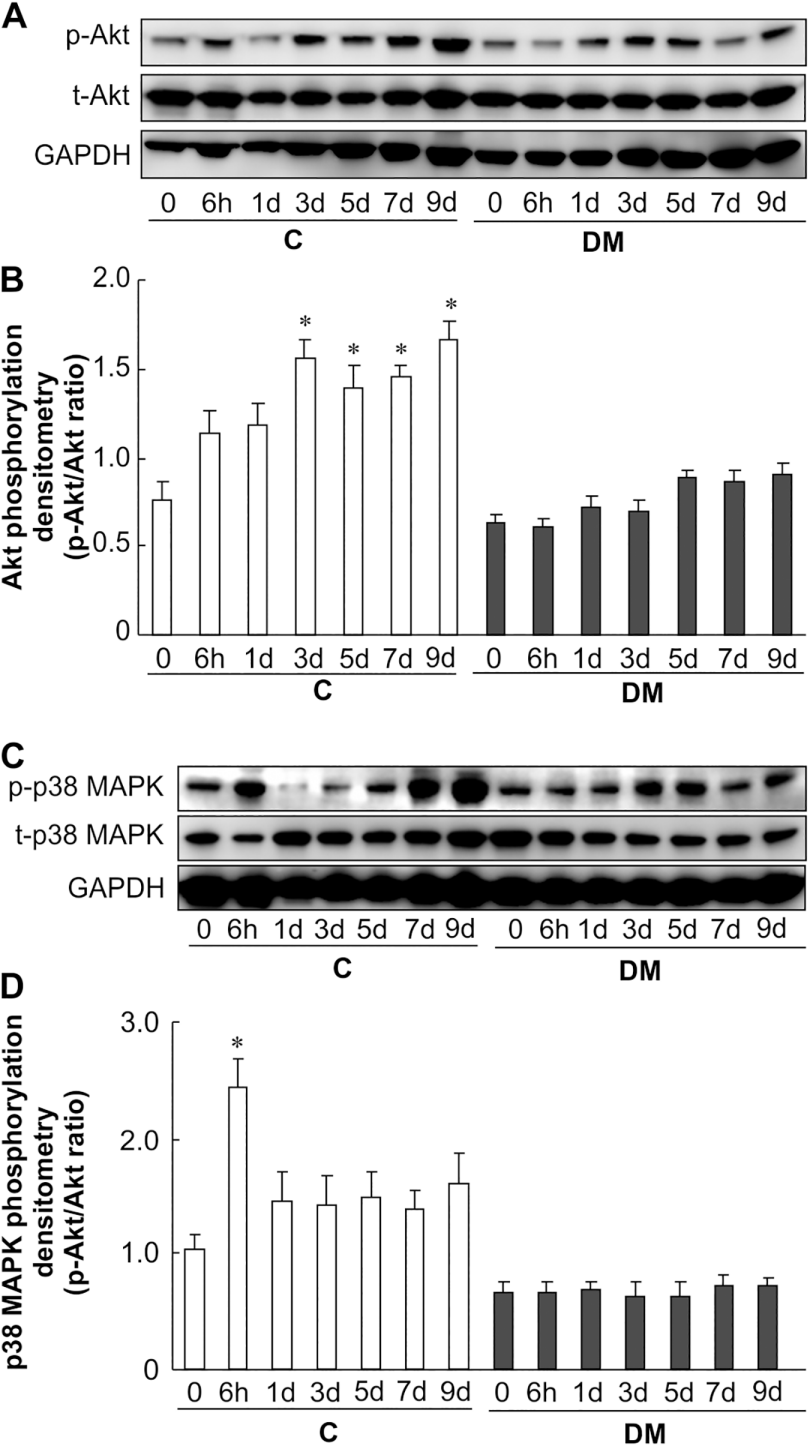
Akt and p38 MAPK activity after skin wounding. (A), (B) Phosphorylation of Akt was analyzed at 0–9 days after wounding in non-diabetic and diabetic mice. Skin lysates were analyzed by western blotting using anti-phospho-Akt and anti-Akt antibodies. (C), (D) Phosphorylation of p38 MAPK was analyzed at 0–9 days in non-diabetic and diabetic mice after wounding. Skin lysates were analyzed by western blotting using anti-phospho-p38 MAPK and anti-p38 MAPK antibodies. The data represent the mean ± SE (n = 6, *p < 0.05 vs. Con (0 h)).

To determine the role of kinases in impaired diabetic wound healing, we analyzed key kinases associated with the regulation of wound healing. Non-diabetic wound skin exhibited increased phosphorylation of Akt at Ser^473^ by approximately 2.5 fold at 3–9 days, but this increase was absent in diabetic wounds (Fig 4A and 4B). In non-diabetic wounds, the phosphorylation of p38 MAPK was approximately 2.5-fold higher at 6 h after wounding. Again, this was not observed in diabetic wound skin (Fig 4C and 4D). These results implicate the Akt and p38 MAPK pathways in the healing of non-diabetic wounds, and such pathways are absent in diabetic wounds.

### IL-6 stimulates migration by primary skin fibroblasts via the p38 MAPK pathway

**Fig 5:**
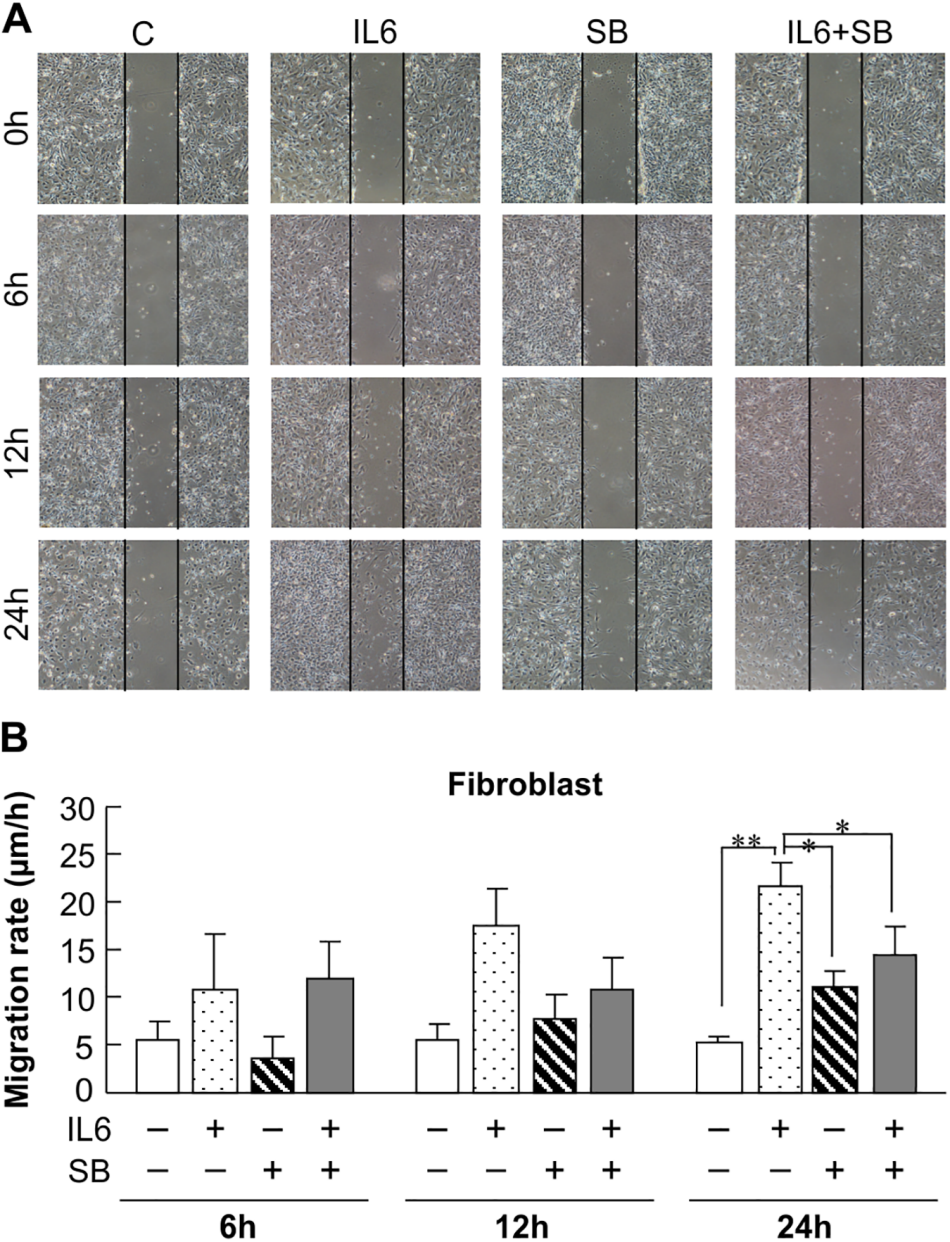
Effect of IL-6 and inhibition of p38 MAPK on fibroblast migration. A: Photos of plates seeded with fibroblasts. Vertical area indicates linear wounds made with a 1-mL pipette tip. Cells were exposed to 10 ng/mL IL-6 with or without pretreatment with 20 μM SB203580 (SB, a p38 MAPK inhibitor) for 0, 6, 12, and 24 h. Vertical scratch width = 500 μm. B: The migration rate of IL-6–stimulated cells treated with or without the p38 MAPK inhibitor was determined by measuring the acellular area at 0, 6, 12, and 24 h. The data represent the mean ± SE of 3 different experiments (*p < 0.05, **p < 0.01 compared with the non-diabetic group).

We performed a scratch assay using primary cultured skin fibroblasts from non-diabetic mice to determine the effect of IL-6 on fibroblast activity. The cells treated with 10 ng/mL IL-6 showed increased migration at 12 h and 24 h compared to the cells not treated with IL-6 (Fig 5A and 5B). Next, fibroblasts were pre-treated with SB203580 (20 μM), a p38 MAPK inhibitor, to test the effect of p38 MAPK signaling on IL-6–induced cell migration. Notably, the IL-6–induced cell migration was significantly suppressed by pre-incubation of the fibroblasts with SB203580 (Fig 5A and 5B). These findings therefore indicate that IL-6 promotes fibroblast migration, and thus wound healing, via the p38 MAPK pathway.

### p38 MAPK, CREB, Akt, and PI3K are activated by IL-6 in primary non-diabetic but not diabetic skin fibroblasts

**Fig 6:**
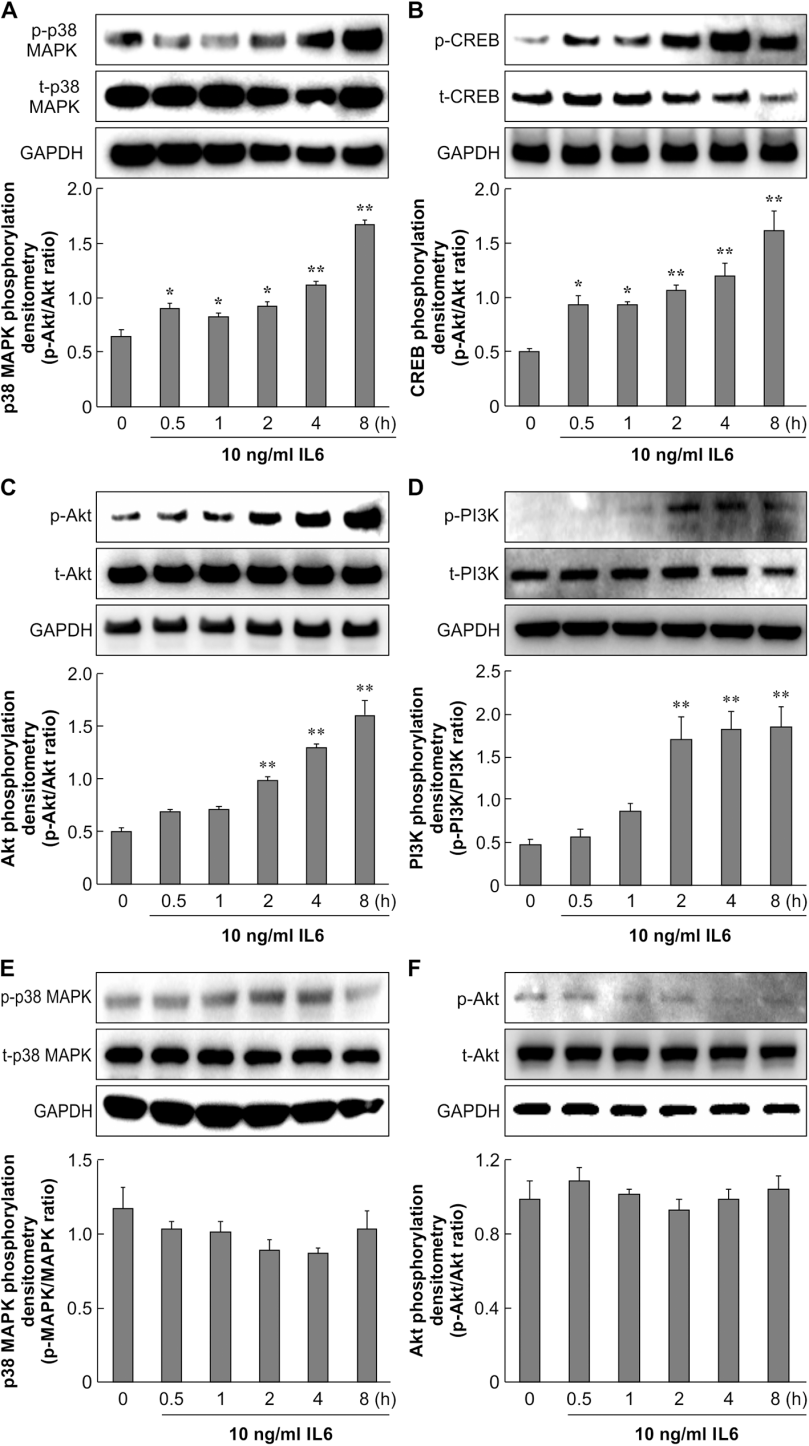
Effect of IL-6 on p38 MAPK and Akt signaling in primary skin fibroblasts. Primary skin fibroblasts from control mice (A, B, C, D) and diabetic mice (E, F) were serum-starved for 16 h. Cells were treated with 10 ng/mL IL-6 and incubated for 0.5, 1, 2, 4, and 8 h. A: Cell lysates were analyzed by western blotting using anti-phospho-p38 MAPK and anti-p38 MAPK antibodies. The data represent the mean ± SE of 3 different experiments (*: *p* < 0.05, **: *p* < 0.01 vs. 0 h). B: Cell lysates were analyzed by western blotting using anti-phospho-CREB and anti-CREB antibodies. The data represent the mean ± SE of 3 different experiments (*: *p* < 0.05, **: *p* < 0.01 vs. 0 h). C, D: Cell lysates were analyzed by western blotting using anti-phospho-Akt, anti-phospho-PI3K, anti-Akt, and anti-PI3K antibodies. The data represent the mean ± SE of 3 different experiments (**: *p* < 0.01 vs. 0 h). E, F: DM cell lysates were analyzed by western blotting using anti-phospho-p38 MAPK, anti-phospho-Akt, anti-p38 MAPK, and anti-Akt antibodies. The data represent the mean ± SE of 3 different experiments.

We identified IL-6 to play an important role in wound healing in the early stage. We next examined the target molecules of IL-6. Akt and p38 MAPK are regulatory kinases that appear to promote the migration of fibroblasts and respond to inflammation [27, 28]. To confirm the role of IL-6 in p38 MAPK and Akt signaling in fibroblasts, we analyzed the phosphorylation of p38 MAPK and its downstream factor CREB, as well as Akt and its upstream factor PI3K. Treatment of non-diabetic skin fibroblasts with 10 ng/mL IL-6 activated phospho-p38 MAPK at 30 min after application, and the phosphorylation of CREB significantly increased from 30 min to 8 h after treatment (Fig 6A and 6B). As shown in Fig 6C and 6D, the level of Akt phosphorylation increased by approximately 2–3 fold with 10 ng/mL IL-6 treatment for 2–8 h. The upstream protein PI3K was also upregulated over time in non-diabetic skin fibroblasts. IL-6 treatment of primary cultured skin fibroblasts from diabetic mice did not induce phosphorylation of p38 MAPK or Akt (Fig 6E and 6F). These data indicate that IL-6-activated phosphorylation of p38 MAPK and Akt was absent in diabetic skin fibroblasts.

### SB203580 attenuates IL-6–stimulated phosphorylation of Akt

**Fig 7:**
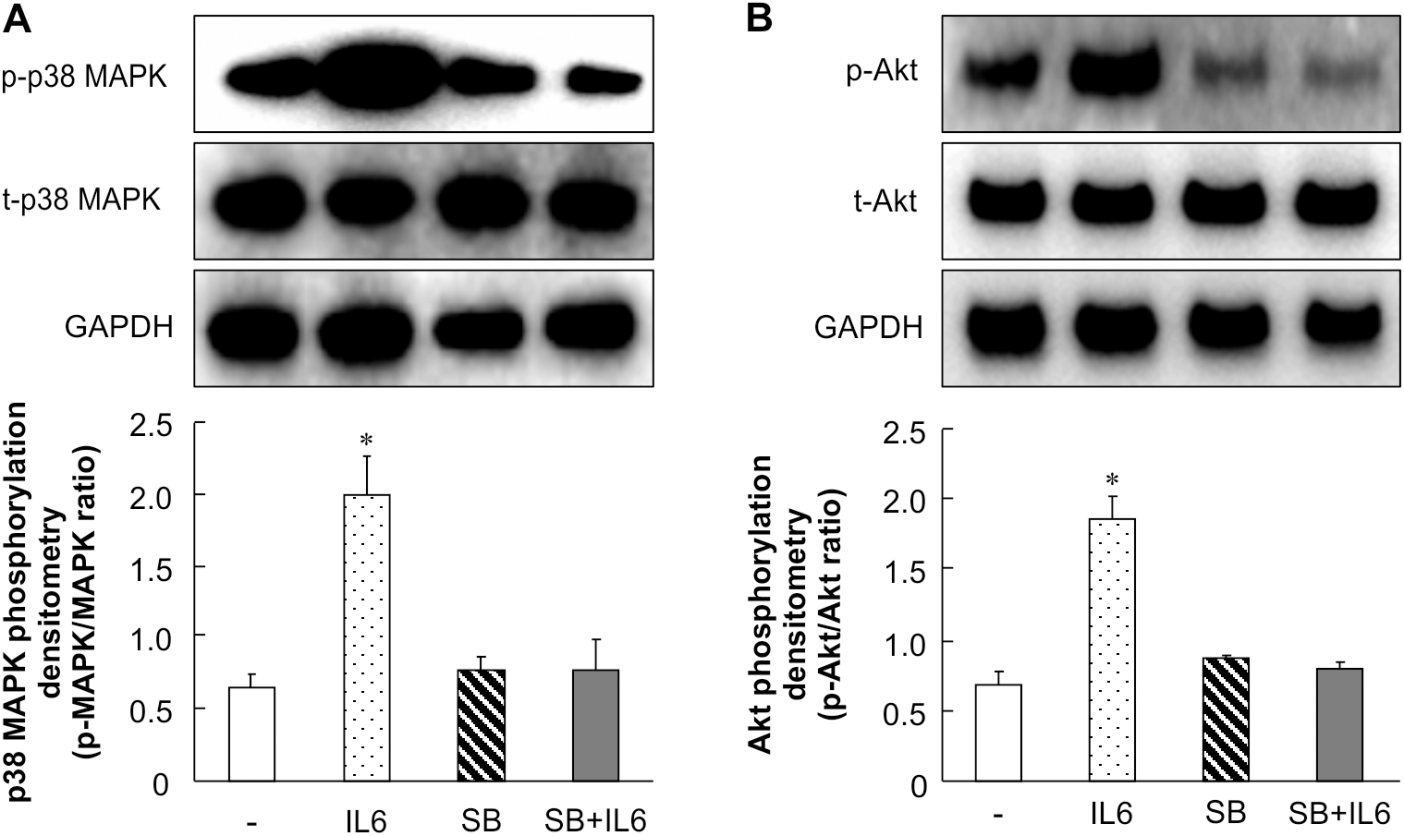
Effect of IL-6 and inhibition of p38 MAPK and Akt phosphorylation in primary skin fibroblasts. Primary skin fibroblasts were serum-starved for 16 h. Cells were treated with 10 ng/mL IL-6 and incubated for 0.5, 1, 2, 4, and 8 h. For inhibitor treatment, primary skin fibroblasts were pretreated with 20 μM SB203580 for 1 h, after which the medium was changed to fresh medium containing 10 ng/mL IL-6. The cells were then incubated for another 8 h. A: Cell lysates were analyzed by western blotting using anti-phospho-p38 MAPK and anti-p38 MAPK antibodies. The data represent the mean ± SE of 3 different experiments (* *p* < 0.05). B: Cell lysates were analyzed by western blotting using anti-phospho-Akt and anti-Akt antibodies. The data represent the mean ± SE of 3 different experiments (* *p* < 0.05).

To further confirm the function of p38 MAPK in wound healing, a p38 MAPK inhibitor was used to suppress the effect of IL-6. As shown in Fig 7A, when fibroblasts were pre-treated with 20 μM SB203580, a p38 MAPK inhibitor, IL-6–stimulated phosphorylation of p38 MAPK was significantly blocked. The IL-6–stimulated phosphorylation of Akt was also attenuated by SB203580 (Fig 7B). These observations imply that p38 MAPK mediates the effect of IL-6 on signaling pathways in a p38 MAPK-Akt–dependent manner.

## Discussion

**Fig 8:**
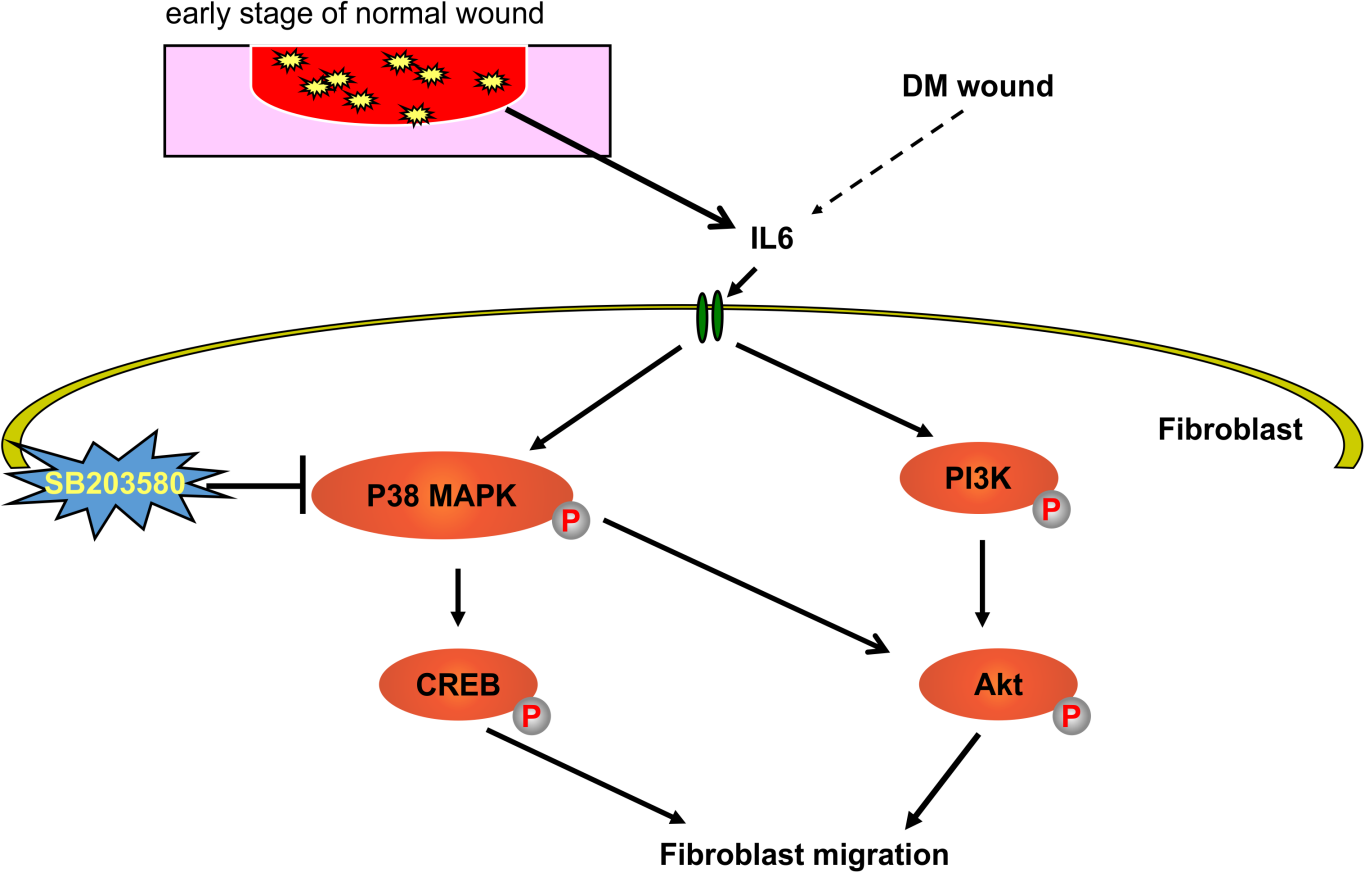
Outline of the mechanisms for wound healing. Using primary skin fibroblasts, we demonstrated that IL-6 stimulates the p38 MAPK-Akt signaling pathway and promotes fibroblast migration. SB203580 blocked IL-6–induced migration of primary control-derived cultured fibroblasts, confirming that IL-6–stimulated fibroblast activity is modulated via the p38 MAPK signaling pathway.

Refractory diabetic wounds are characterized by a persistent inflammatory response, leading to impaired progression of the healing process [3, 11, 12, 16]. This study shows that the macrophage polarization status and expression of IL-6 significantly differ between non-diabetic and diabetic mice during pre-injury and in the early phase of wound healing. Our results provide the first evidence that IL-6 stimulates the migration of non-diabetic fibroblasts through activation of the p38 MAPK and PI3K/Akt signaling pathways (Fig 8); however, in diabetic fibroblasts, these signaling pathways are dysfunctional.

Macrophage depletion and/or aberrant activation are known to result in impaired wound healing [12, 16, 34]. In diabetic wounds, we noted significantly increased expression of the macrophage marker CD68 at almost all time points, ruling out a role for macrophage depletion–associated impairment in diabetic wound repair [35]. However, the inflammatory markers iNOS, IL-6, and TNF-α were upregulated in pre-injury diabetic skin, suggesting persistent inflammation. Interestingly, we also noted increased mRNA expression of the anti-inflammatory cytokines IL-10, IL-4, and TGFβ in pre-injury skin of diabetic mice. Such an increase indicates the presence of a compensatory but futile counter-regulation of proinflammatory stimuli, as postulated by Herder et al. [36]. Non-diabetic mice also showed significantly higher expression levels of Arg-1 mRNA at several time points than diabetic mice. Furthermore, increased expression of Arg-1, an M2-related gene, appeared earlier in the non-diabetic mice compared to the diabetic mice, suggesting delayed activation of the M2 phenotype in diabetic wounds. Taken together, these results confirm that post injury, the temporal cytokine profile considerably differs between diabetic and non-diabetic wounds, and that M1/M2 macrophage polarity is skewed in favor of M1 in diabetes [12, 37, 38].

At 6 h post-wounding, the expression of IL-6 was significantly higher in non-diabetic wounds than in diabetic wounds. The suppressed expression of IL-6, an M1-related inflammatory cytokine, reflects the abnormal initiation of the inflammatory phase in diabetic wounds. IL-6 regulates the hypothalamic-pituitary-adrenal axis and is involved in monocyte chemotaxis, angiogenesis, and collagen accumulation, which are critical for wound regeneration [39–42]. Therefore, reduced IL-6 levels in the early stage of wound healing have the potential to adversely affect the subsequent phases of wound healing, including the activation of macrophages [43, 44]. Furthermore, because the diabetic wound site is chronically exposed to higher IL-6 levels, the IL-6 receptor (IL-6R) response might be attenuated, desensitizing cells to the post-wounding increase in IL-6 levels [41, 45]. All of these factors strongly indicate that pre-injury chronic exposure to IL-6 and low abundance of IL-6 post injury both contribute to delayed wound healing [44, 46, 47]. However, further research is needed to fully establish why the balance between proinflammatory and anti-inflammatory cytokines is shifted toward proinflammation in diabetes and whether the chronic proinflammatory environment in diabetes affects cytokine function in cell signal transduction, IL-6R response, macrophage activation, and chemotaxis [37, 38, 48, 49].

Phosphorylation of Akt and p38 MAPK was observed in the non-diabetic but not diabetic wounds. We investigated the role of IL-6 in stimulating the signaling pathways of the protein kinases PI3K/Akt and stress-activated protein kinase p38 MAPK in diabetic and non-diabetic fibroblasts. In non-diabetic fibroblasts, IL-6 increased the phosphorylation of p38 MAPK and the levels of its downstream factor CREB. It also significantly increased Akt phosphorylation and the levels of its upstream factor P13K. These effects of IL-6 were not detected in diabetic fibroblasts. Furthermore, in scratch assays, we found that IL-6 stimulated fibroblast migration and that this IL-6-stimulated migration was blocked by the selective p38 MAPK inhibitor SB203580, confirming the involvement of p38 MAPK pathways in non-diabetic fibroblast activity. MAPKs are known to be involved in inflammation, cell proliferation, migration, and differentiation, and several reports have confirmed the role of the p38 pathway in influencing the migration of different cell types [50–56]. Our study therefore demonstrates that IL-6-induced fibroblast migration requires p38 MAPK and Akt activation, and that this effect is impaired in diabetic fibroblasts.

In conclusion, our study demonstrated for the first time that diabetic wounds show considerably abnormal temporal cytokine profiles. In particular, 6 h post injury, IL-6 mRNA expression was significantly lower in diabetic wounds. Abnormal macrophage activation, but not the lower abundance of CD68-expressing macrophages, was found to be characteristic of diabetic wounds. IL-6 played a key role in facilitating non-diabetic fibroblast migration during wound healing via the p38 MAPK-Akt pathway. This signaling pathway was found to be dysfunctional in diabetic fibroblasts/wounds. Taken together, our results indicate that the regulation of IL-6 activity, functioning of p38 MAPK signaling pathways, and resolution of impaired macrophage activation should be considered when developing therapeutic strategies for non-healing diabetic wounds.

## Supporting information

S1 Fig Positive and negative immunostaining for Arg-1 (green). Nuclear counterstaining was performed using DAPI (blue). Scale bar = 100 μm. Liver tissue sections were stained with Arg-1 antibodies. Negative controls lacked the primary antibody for Arg-1.
(TIF)

S2 Fig CD68 staining of Mouse spleen tissue. **Positive and negative immunostaining for CD68 (red).** Nuclear counterstaining was performed using DAPI (blue). Scale bar = 100 μm. Spleen tissue sections were stained with CD68 antibodies. Negative controls lacked the primary antibody for CD68.
(TIF)
