# Supplementary figures and images for: Interleukin-6 stimulates Akt and p38 MAPK phosphorylation and fibroblast migration in non-diabetic but not diabetic mice
Source: PLoS One. 2017 May 23;12(5):e0178232. doi: 10.1371/journal.pone.0178232 (PMC5441644; doi:10.1371/journal.pone.0178232)

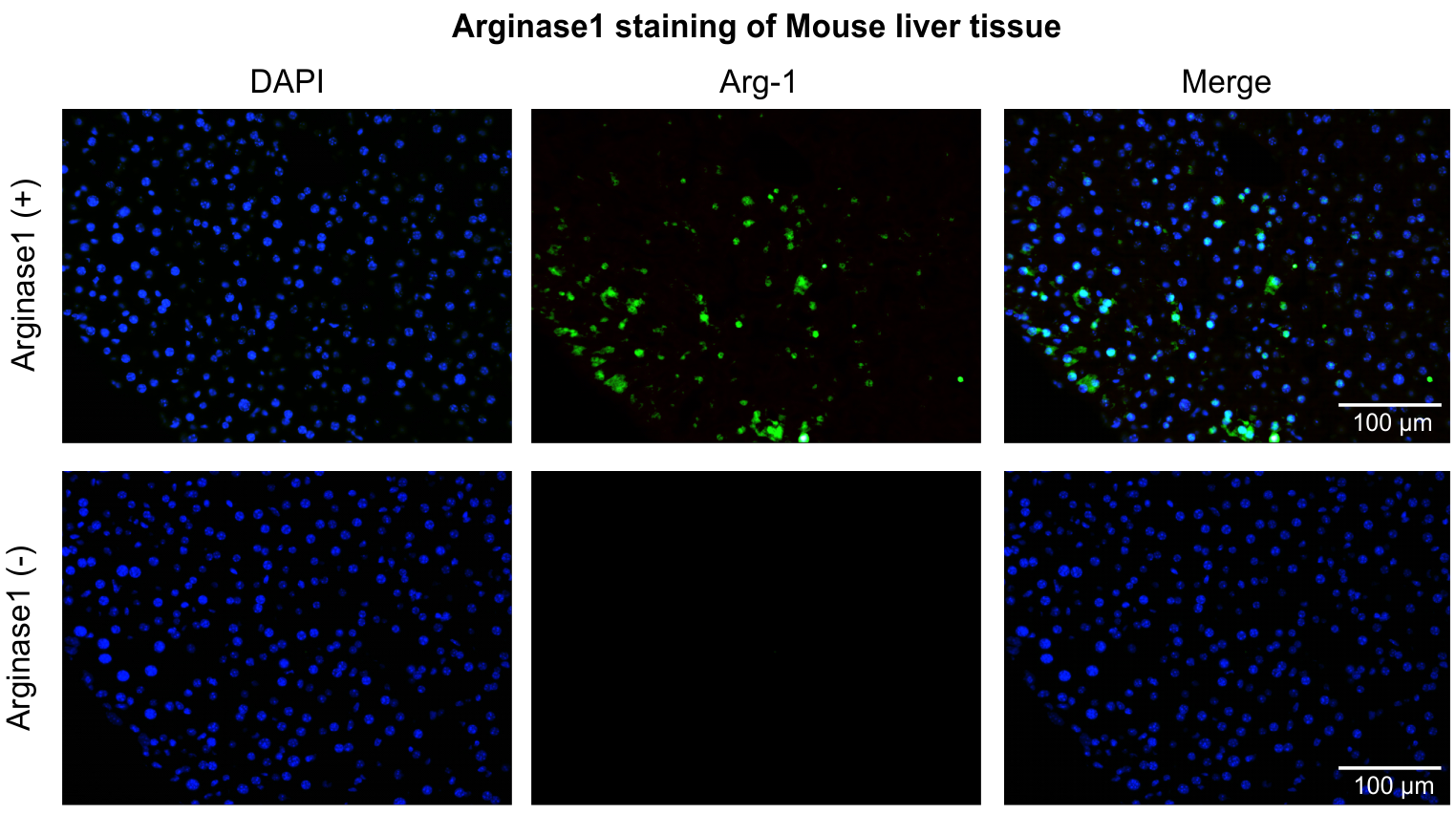

Supplement: S1 Fig — Nuclear counterstaining was performed using DAPI (blue). Scale bar = 100 μm. Liver tissue sections were stained with Arg-1 antibodies. Negative controls lacked the primary antibody for Arg-1. (TIF) [file pone.0178232.s001.tif]

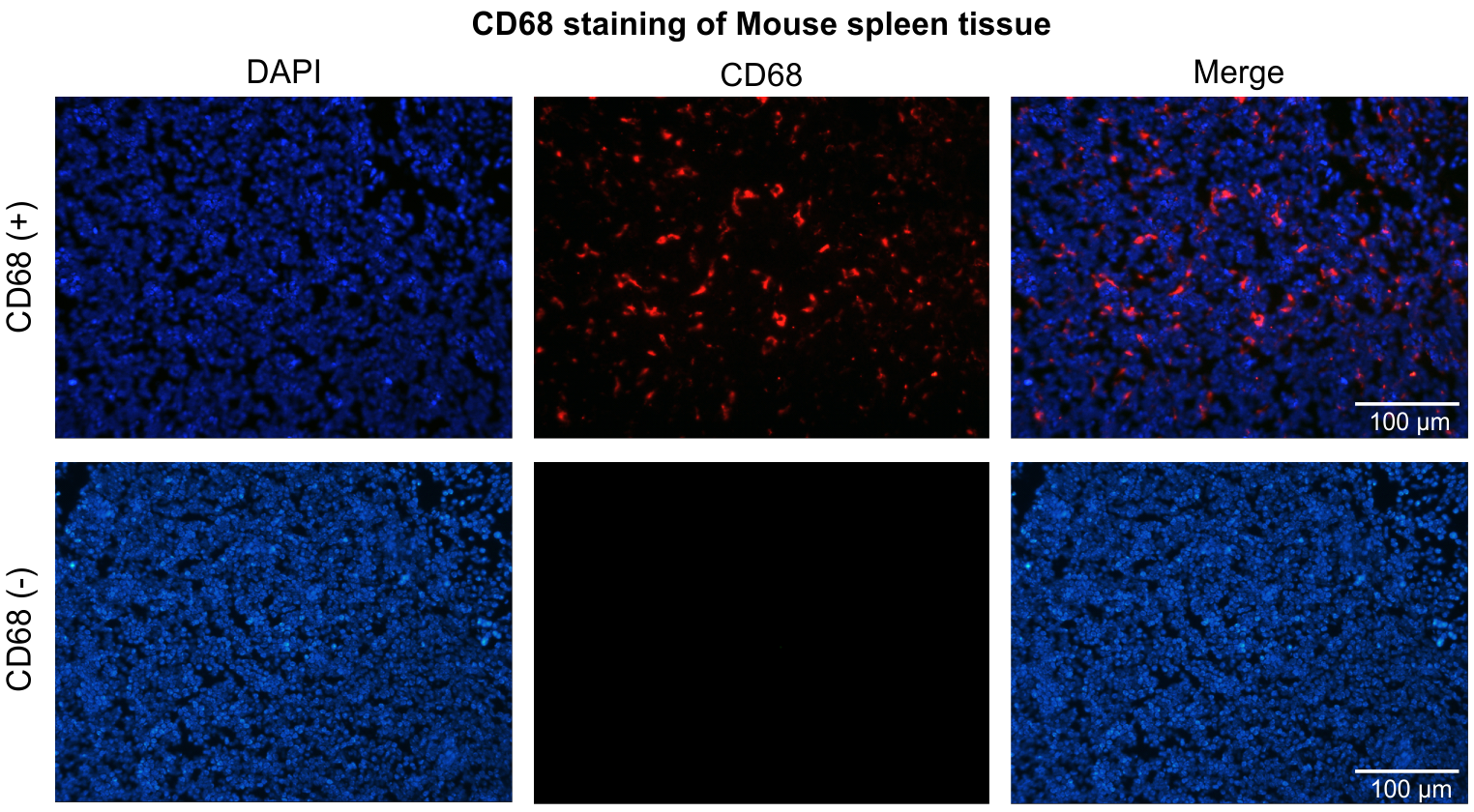

Supplement: S2 Fig — Positive and negative immunostaining for CD68 (red). Nuclear counterstaining was performed using DAPI (blue). Scale bar = 100 μm. Spleen tissue sections were stained with CD68 antibodies. Negative controls lacked the primary antibody for CD68. (TIF) [file pone.0178232.s002.tif]
